# Human domestication and the roles of human agency in human evolution

**DOI:** 10.1007/s40656-020-00315-0

**Published:** 2020-05-26

**Authors:** Lorenzo Del Savio, Matteo Mameli

**Affiliations:** 1 Ludwig-Maximilians-Universität München, Munich, Germany; 2 Department of Philosophy, King’s College London, London, United Kingdom

**Keywords:** Domestication, Human evolution, Niche construction, Agency, Violence, Political selection

## Abstract

Are humans a domesticated species? How is this issue related to debates on the roles of human agency in human evolution? This article discusses four views on human domestication: (1) Darwin’s view; (2) the view of those who link human domestication to anthropogenic niche construction and, more specifically, to sedentism; (3) the view of those who link human domestication to selection against aggression and the *domestication syndrome*; and (4) a novel view according to which human domestication can be conceived of in terms of a process of *political selection*. The article examines and compares these views to illustrate how discussions of human domestication can contribute to debates about how, and to what extent, human agency has affected human evolution.

## Introduction

Are humans domesticated? In *The Descent of Man*, Charles Darwin argued that humans are not a domesticated species. Darwin’s treatment of this topic was a response to claims made by Johann Friedrich Blumenbach, who, at the end of the 18th century, amidst discussions of civilization and domestication, had written that humans are “far more domesticated and far more advanced than other animals” (Blumenbach [1795] 1865, p. 205; Darwin 1871, p. 111). Using Darwinian arguments, Walter Bagehot defended the human domestication hypothesis in a book published the year after *The Descent of Man*. According to Bagehot, human domestication constituted the foundation of human civilization (Bagehot 1872). In the first decades of the 20th century, the thesis that some human groups (or “races”), but not others, are domesticated was used to support racist policies and eugenic projects (Brüne 2007). Some scholars opposed this thesis by claiming that all extant humans are domesticated (Boas 1911; cf. also Mead 1954). In 1962, Theodosius Dobzhansky wrote that the notion of human domestication was “too vague an idea to be scientifically useful” (Dobzhansky 1962, p. 196). Recently, the idea that humans have undergone a process of domestication and that this was crucial for the evolution of our species has re-emerged. Attempts have been made to find useful ways of eliminating the vagueness surrounding the notion of human domestication.^1^

This article neither aims to identify the correct way of conceiving of human domestication, nor aims at policing the terminology used by empirical researchers when they investigate this topic. Instead, the article reveals the relevance of discussions of human domestication for debates about how, and to what extent, human agency has had an impact on human evolution. Section 2 discusses Darwin’s views on human domestication and their connection with his views on what he calls “man’s selection” (Darwin 1868, 1871). Section 3 discusses the views that link human domestication to the selective impact produced by human activities when sedentism spread (cf. Leach 2003). Section 4 explores the thesis that human domestication was produced by endogenous selection against aggression and resulted in a human version of what is known as the “domestication syndrome” (cf. Wrangham 2019; Belyaev 1979). Section 5 argues that an additional conception of human domestication can be formulated in terms of a process of *political selection*: humans may have become domesticated as a result of their political agency.

All four of the ways of thinking about human domestication discussed in the article are interesting and useful. This is an area where conceptual mismatches have been productive. Different conceptions of what human domestication involves—and different hypotheses about whether it has actually occurred—provide access to distinct (but not incompatible) ideas about specific ways in which human agency might have affected the evolutionary trajectory of the human species.^2^

## Human domestication and artificial selection according to Darwin

As is well known, in *On the Origin of Species* Darwin argues for the biological possibility of evolution by means of natural selection through an analogy with artificial selection, which he calls “man’s selection” (cf. Darwin 1859, Chapter 4). Darwin distinguishes two kinds of artificial selection: “methodical” and “unconscious.” In *Variations of Animals and Plants under Domestication*, this distinction is made in these terms:

Selection may be followed either methodically and intentionally, or unconsciously and unintentionally. Man may select and preserve each successive variation, with the distinct intention of improving and altering a breed, in accordance with a preconceived idea; and by thus adding up variations, often so slight as to be imperceptible by an uneducated eye, he has effected wonderful changes and improvements. It can, also, be clearly shown that man, without any intention or thought of improving the breed, by preserving in each successive generation the individuals which he prizes most, and by destroying the worthless individuals, slowly, though surely, induces great changes (Darwin 1868, Vol. I, pp. 3–4).

The two varieties of artificial selection identified in the above quote require distinct activities on the part of a human selecting agent. Methodical selection occurs when a human selecting agent operates with the purpose of producing specific results (practical or aesthetic) in a lineage. In contrast, unconscious selection is brought about by human selecting agents acting on their preferences for traits that they find useful or beautiful, but without any intent to produce a change in the lineage. “Man’s selection”, in its two varieties, always involves a choice on the part of the selecting agent: a choice to allow (or encourage or coerce) some variants—the “prized” ones—to reproduce, to the detriment of other variants. The only difference between methodical and unconscious selection is that in methodical selection the choice is made with the goal of obtaining some specific changes in future generations, while in unconscious selection it is not.

In Darwin’s view, methodical and unconscious selection are the only two domesticating processes; no other process generates domestication. The domesticating agent is the human agent who artificially selects some variants over others by making choices of the kind just mentioned.^3^ In his book on domestication, Darwin discusses the domestication of dogs, cats, horses, asses, pigs, cattle, sheep, goats, rabbits, pigeons, fowls, ducks, geese, turkeys, guineafowls, canary birds, gold fish, hive bees, and silk moths, as well as various plants. Darwin describes some recurring features of domesticated populations that set them apart from wild ones. He notices that domesticated populations often have, relative to their non-domesticated predecessors, higher interspecific variability (Darwin 1868, Vol. II, p. 250), higher fertility (Darwin 1868, Vol. II, p. 111), and—in the case of some vertebrates—smaller skulls and brains (Darwin 1868, Vol. II, p. 298). For Darwin, the cross-species similarities and characteristic outcomes produced by domestication are interesting because they can be used as fallible ways of detecting the occurrence of domestication. However, these similarities are not part of the real definition of domestication. In his view, domestication *is* “man’s selection.”

Given Darwin’s general views on domestication, what is his opinion on human domestication? As stated above, in *The Descent of Man* he rejects the hypothesis that humans are domesticated (Darwin 1871, Vol. I, p. 111). He concedes that the human species shares some interesting features with species known to be domesticated. He mentions the high fertility observed in “civilised nations” (Darwin 1871, Vol. I, pp. 132–133) and also the similarities between the intraspecific variability of human beings and that of domesticated non-human animals (Darwin 1871, Vol. I, p. 111).^4^ However, these similarities between the human species and species known to have been artificially selected are insufficient to demonstrate that humans have been artificially selected, either methodically or unconsciously. If humans have not been artificially selected, they cannot be domesticated.

Darwin argues that artificial selection is a rare phenomenon within human populations:

Man differs widely from any strictly domesticated animal; for his breeding has never long been controlled, either by methodical or unconscious selection. No race or body of men has been so completely subjugated by other men, as that certain individuals should be preserved, and thus unconsciously selected, from somehow excelling in utility to their masters. Nor have certain male and female individuals been intentionally picked out and matched, except in the well-known case of the Prussian grenadiers; and in this case man obeyed, as might have been expected, the law of methodical selection; for it is asserted that many tall men were reared in the villages inhabited by the grenadiers and their tall wives (Darwin 1871, Vol. I, p. 111).

Darwin recognizes that some attempts at methodical selection have taken place within human populations. For example, Frederick William I of Prussia coerced many tall men to join his Potsdam Giants regiment and also coerced many tall women to marry and have children with these men. The Soldier King, as he was known, wanted to create a regiment of taller than average grenadiers and acted on his views about how height is inherited. However, the plan was small scale and short lived, so it did not produce any significant evolutionary change.^5^

According to Darwin, a theory of domestication is a theory of “the amount and nature of the changes” that organisms “undergo whilst under man’s dominion” (Darwin 1868, Vol. I, p. 1). Slavery can result in some individuals becoming “completely subjugated by other men,” including in terms of their reproductive activities. Could this not produce some form of artificial selection (methodical or unconscious) within the human species? Darwin, who as a fervent abolitionist had written on the horrors of slavery (cf. Desmond and Moore 2011), does not discuss this issue. One plausible hypothesis is that, just like for the Prussian case, he does not think that the choices of slave owners in relation to the reproduction of their most “prized” slaves could lead to evolutionarily significant changes.^6^

Darwin occasionally suggests that sexual selection among humans is a form of unconscious selection (Darwin 1871, Vol. II, pp. 369–371; cf. Alter 2007). Sexual selection occurs when the members of one sex choose preferentially to mate with opposite-sex organisms with certain “prized” traits. According to Darwin (1871), sexual selection in humans is driven primarily by male competition and male choice: the “victorious” men, by choosing for themselves the most “prized” women, determine who is going to mate and with whom.^7^ These choices are not (in general) aimed at changing the lineage, but they do change it. This is why Darwin links sexual selection to unconscious selection rather than methodical selection. However, if sexual selection in humans is a kind of unconscious selection, and if unconscious selection is a variety of “man’s selection,” the question of why Darwin does not consider the possibility that humans have become domesticated through sexual selection arises.^8^ One hypothesis is that he does not consider this because he believes that, despite some similarities, the differences between sexual selection and standard cases of artificial selection speak against an extension of the term “domestication” to circumstances where the driving selective factor is mate choice. Perhaps the idea is that the two kinds of reproductive choices—those made by the “victorious” men and those made by “breeders,” “fanciers,” and “masters,”—operate in different ways; or perhaps the idea is that “man’s dominion” is stronger in the inter-species case than it is in the intra-species interactions between men and women. Again, Darwin does not discuss the issue.

Darwin’s claim that “man’s selection” of humans by humans, in its two possible varieties, is a rare phenomenon is certainly plausible *if* slavery and sexual selection are put aside. Within such a framework, equating domestication with “man’s selection” leads to the view that humans are not domesticated. Darwin’s opinion on this issue is influential; it is the orthodox view. However, in recent discussions the Darwinian conception of domestication as artificial selection has been abandoned. “Man’s selection” is no longer considered necessary for domestication. Artificial selection involves a particular and peculiar form of human agency: choosing whether to allow (or coerce) some other organism (of one’s own or another species) to reproduce. Whether this form of human agency has had an impact on human evolution—for example through slavery or mate choice—is an interesting issue. However, once one relinquishes the idea that this form of human agency is required for domestication, debates on human domestication can focus on the evolutionary consequences of other forms of human agency.

## Human domestication, niche construction, and sedentism

Human choices and activities can alter the selective environments in which human and non-human populations evolve. They can also alter the ontogenetic environments in which human and non-human organisms develop. All these processes can be said to be processes of *anthropogenic* (i.e. human-made) *niche construction*.^9^ Both unconscious and methodical selection are special forms of anthropogenic niche construction. In the vast majority of cases, anthropogenic niche construction produces evolutionary effects that are not the direct result of human choices concerning which members of a given population are allowed to reproduce and which are not.

If one develops a way of thinking according to which domestication does not require artificial selection, it becomes possible to classify humans as domesticated even if Darwin is right that artificial selection of humans by humans is rare, short lived, and evolutionarily marginal. Some scholars have argued that a theory of anthropogenic niche construction can provide an explanation of many of the processes that are normally conceived of in terms of domestication (Zeder 2016; Smith 2016). Is there a theoretically useful way of linking domestication to anthropogenic niche construction?^10^ Relying on the etymology of the term *domestication*, Helen Leach suggests that domestication should be understood as due not to human choices and activities in general but, more narrowly, to those that have to do with the *domus*:

Though the term “domestication” is not universally acceptable, it does serve to draw attention to the potential role of the built environment and other cultural constructions in bringing about […] changes in humans and animals. […] By viewing domestication in its broadest sense as acclimatization to life in a household and extending that concept to incorporate the house and its outbuildings, yards, gardens, and orchards […], we can reconsider some of the criteria of domestication as biological changes brought about through living in this culturally modified, artificial environment. […] A key factor in this human-domestication hypothesis is the artificial protective environment created by humans and shared progressively with animals and plants. (Leach 2003, pp. 359–360; cf. Leach 2007; Wilson 1988, 2007).

In this view, “breeding intentions” and choices concerning which individuals should be allowed to reproduce are not needed for domestication to occur. Domestication does not require artificial selection; rather, it requires artificial environments of the kind that sedentism made possible. The diffusion of sedentism was a turning point in human evolution. Its effects included major dietary and epidemiological shifts, changes in the patterns of physical activity, and microclimate modifications. The traits of humans, and of various species with which humans interacted, were transformed. In Leach’s view, all these species can be said to be domesticated as a result of these transformations.^11^

The view according to which domestication is an evolutionary consequence of permanent human settlements can help us make sense of some claims found in the literature. For example, some researchers classify the house mouse (*Mus musculus*) as a domesticated species (Boursot et al. 1993; Weissbrod et al. 2017; Geiger et al. 2018). The house mouse has not been artificially selected, either methodically or unconsciously: the ways in which house mice differ from wild murids are not the result of methodical breeding, nor, more generally, are they the result of humans choosing to allow the reproduction of “prized” mice to the detriment of those that are not “prized.” Human preferences regarding the reproduction of different variants within a species always play a direct role in the two varieties of “man’s selection” discussed by Darwin. However, in the case of *Mus musculus*, the phenotypic changes are simply the selective by-products of the proximity between humans and mice and of the stable ways in which humans have modified their surroundings through permanent settlements. Within Leach’s framework, this is sufficient for saying that *Mus musculus* is a domesticated species.^12^

Leach’s view also allows us to understand claims on the domestication of microbes such as yeasts. Nowadays, many microbes that play a role in human food production are the target of methodical selection, but researchers (e.g. Libkind et al. 2011; Steensels et al. 2019) argue that the domestication of these microorganisms—those used in the production of bread or beer, for example—started thousands of years ago. Various processes of food production that became possible after the adoption of sedentism generated selection pressures that transformed these microbes. In general, the variants that better contributed to these processes of food production were more evolutionarily successful. However, this was not the result of “man’s selection” in Darwin’s sense. For thousands of years, humans did not know about the existence of these microorganisms. Thus, humans could not make choices about which variants (the “prized” ones) should be allowed to reproduce and which variants should not. Nevertheless, these microbes—which have become “acclimatized” to the *domus*—count as domesticated in Leach’s sense.^13^

On a view of domestication like Leach’s, domestication can occur when “man’s selection” is absent, or before it kicks in. In both Darwin’s account and Leach’s account, domestication is due to human agency. However, Darwin’s account focuses on the direct effects of human choices concerning which variants are allowed to reproduce and which ones are not, while Leach’s account focuses on the direct and indirect effects of stable human-made environments. It is because humans have been evolutionarily transformed by human permanent settlements that Leach proposes to regard humans as a domesticated species.

Both Darwin’s theory of domestication as artificial selection and Leach’s theory of domestication as resulting from human permanent settlements are interesting. When applied to the issue of human domestication, Darwin’s account and Leach’s account allow one to explore two different ways in which human agency might have affected human evolution. However, there are other ways of conceiving of human domestication that can generate further insights into the evolutionary roles of human agency.

## Human domestication, the domestication syndrome, and selection against aggression

Darwin observes that domesticated species sometimes differ from their wild ancestors in similar ways. This has led some researchers to hypothesize that it might be useful to identify traits shared by domesticates, at least within specific classes of organisms. Mammalian domesticates have been observed to have traits such as floppy or reduced ears, shorter muzzles, curly tails, smaller teeth, smaller cranial capacity (and concomitant brain size reduction), more frequent estrous cycles, some depigmentation, pedomorphosis, neotenous behavior and increased social tolerance, reduction of sexual dimorphism (feminization) and increased docility (Theofanopoulou et al. 2017). These traits have been said to constitute a mammalian *domestication syndrome*.^14^ On the basis of a comparison between extant humans and the inferred features of our Lower Paleolithic ancestors, Richard Wrangham and colleagues argue that the mammalian domestication syndrome is also present in humans (Wrangham 2019; Cieri et al. 2014).^15^ Modern humans display a number of morphological, physiological, developmental, behavioral, and cognitive features that, according to these authors, can be seen as manifestations of a human version of the mammalian domestication syndrome:

### *Anatomy*:

Feminized faces, globular cranial development, depigmentation of the sclera, modest reduction in cranial/brain size;

### *Physiology*:

Reductions in neonatal androgens and pubertal testosterone levels, increased brain serotonin and oxytocin availability;

### *Development*:

Graded brain development with extreme delays of synaptic pruning, early-emerging social cognition;

### *Behavior*:

Increased social tolerance, food sharing, helping, social bonding;

### *Cognition*:

Cooperative communication, cumulative cultural evolution, expanded social networks.^16^

On the basis of a comparison between observed bonobo traits and the inferred traits of the *Pan* common ancestor, Wrangham and colleagues argue that the mammalian domestication syndrome is also present in bonobos (cf. Hare 2017; Hare and Wrangham 2017). Bonobos are not normally classified as a domesticated species, which raises the question: how could bonobos have the domestication syndrome? Bonobos have not been the target of artificial selection and they have not been shaped by an association with human permanent settlements. If one wants to classify bonobos as a domesticated species, one needs to adopt a view of domestication according to which domestication does not require “man’s selection” and, more generally, according to which it does not require anthropogenic niche construction. For bonobos to be a domesticated species, it must be possible for domestication to occur without human agency being involved. So, what is the account proposed by Wrangham?

The mammalian domestication syndrome was observed in the silver foxes selected as part of an experiment that Dmitri Belyaev began at the end of the 1950s and was then continued by his collaborator, Lyudmila Trut, after his death in 1985 (Trut 1999; Hare et al. 2005; cf. also Dugatkin and Trut 2017; Hare and Woods 2013). In this study, the foxes were artificially selected exclusively for reduced levels of aggression toward human keepers. According to Belyaev and Trut, in a small number of generations the foxes developed many of the traits found in the mammalian domestication syndrome (cf. Belyaev 1979; Trut 1999). Building on this work, Wrangham argues that selection against aggression is the domesticating process. On this view, selection against aggression is the evolutionary process that produced the domestication syndrome in all the mammalian species that have it.^17^

The view that animal domestication and selection for tameness are linked is an old one.^18^ If some animals are not docile, human beings are unlikely to find them useful, or allow them in proximity of their houses (cf. Galton 1865). Thus, selection for tameness is—at least indirectly—involved both in Darwin’s “man’s selection” and in Leach’s “acclimatization” to the *domus*. However, diverging from both Darwin and Leach, Wrangham makes selection for tameness the essential ingredient in his conception of domestication. In terms of the Belyaev–Wrangham framework, any species—including humans and bonobos—can be domesticated if there is selection against aggression, independent of any role played by artificial selection, human permanent settlements, or human agency more generally.

In one of his articles, Belyaev hypothesizes that selection against aggression has had an important impact on human evolution: “the anthropogenesis is surprisingly similar to what is observed in animal domestication [i.e., in the silver foxes]” (Belyaev 1984, p. 383). According to Belyaev, selection against aggression in human populations is a by-product of the human social (and socially constructed) niche:

The selection required from the individuals some new properties: ability of obedience to the requirements and traditions of the society and concrete social environment, i.e. self-control in social behavior and self-estimation. It is precisely under these conditions that the biosocial nature of man and biosocial function of his brain was consolidated. The social environment created by man himself has become for him quite a new ecological milieu. (Belyaev 1984, p. 385).^19^

Using Christopher Boehm’s work, Wrangham attempts to identify what aspects of the human social niche could have generated the relevant kind of selection pressures (cf. Wrangham 2019; Boehm 1993, 2001, 2012). According to Boehm, the small-scale hunter-gatherer societies most representative of the ways of life of our Late Pleistocenic ancestors have *reverse dominance hierarchies*. In closely related species, such as chimpanzees and gorillas, alpha males dominate the whole troop. In contrast, would-be alphas in hunter-gatherer bands—that is, individuals who would like to command and control the whole group—are systematically defeated by the rank and file. The potential subordinates, by forming a robust alliance, find ways to punish the would-be dominators and enforce a form of egalitarianism.^20^ Violent individuals who try to impose their will on the group (in order to obtain a greater share of resources) are punished, according to Boehm. Some forms of punishments—such as ridicule—are mild, but others are harsh and can result in expulsion from the group or even in execution and death (capital punishment). Anti-alpha coalitions have occasionally been observed in captive apes, but they are rare, fragile, and short lived (e.g. de Waal 1996, pp. 91–92). According to Boehm, in Late Pleistocenic human populations, anti-alpha coalitions became common, robust, long lived; because of this, they became capable of producing evolutionarily significant effects.

Wrangham claims that coalitionary behaviors like those described by Boehm, and especially the coordinated killings of alpha-type individuals, produced selection pressures against aggression in humans. According to this view, humans have become domesticated as a result of these coalitions and these executions. Wrangham calls it “the execution hypothesis”: executions produced selection against aggression, which in turn produced the human version of the mammalian domestication syndrome. He observes:

Unfortunately to quantify the past rates of execution, or to calculate the Pleistocene selection pressures, is impossible. The concept that the domestication syndrome results from capital punishment is supported, however, by the human system of male egalitarianism, because the primate-style alpha males that are missing from human society are characteristically reactive aggressors. (Wrangham 2019, p. 160).^21^

Wrangham (2019, p. 129) draws attention to a section in *The Descent of Man* where Darwin discusses the impact that the punishment of criminals might have had on human evolution:

In regard to the moral qualities, some elimination of the worst dispositions is always in progress even in the most civilised nations. Malefactors are executed, or imprisoned for long periods, so that they cannot freely transmit their bad qualities. Melancholic and insane persons are confined, or commit suicide. Violent and quarrelsome men often come to a bloody end (Darwin 1874, Vol. I, p. 137).

Wrangham uses this passage to argue that the intuition behind his “execution hypothesis” is already present in Darwin’s work, even though, as a result of his views on domestication and artificial selection, Darwin makes no connection between executions and the issue of human domestication. The differences between Darwin’s views and Wrangham’s views become evident when one focuses on bonobos.

Wrangham argues that bonobos have become domesticated as a result of selection pressures against aggression (Hare et al. 2012). How did it happen? Humans were not involved. Due to favorable ecological conditions and correlated residency patterns, female bonobos tend to stay together, which facilitates female coalition formation. Coalitions of female bonobos are usually able to neutralize bullying and domineering males in a stable and consistent way, resulting in dominance hierarchies that are different from those observed in chimpanzees, where anti-bullying female coalitions are rarer. Bonobo hierarchies are not reverse dominance hierarchies in Boehm’s sense, but they often have female coalitions at the top, while the more rigid chimpanzee hierarchies are dominated by one or a small number of males and all the females are, in general, subordinated to all the adult males (cf. de Waal 1982; Wrangham and Peterson 1997). Wrangham’s (and Hare’s) hypothesis is that female coalitions were able to generate selection against aggression in bonobo populations, which in turn generated, without any human involvement, a bonobo version of the mammalian domestication syndrome.

In some cases, such as Belyaev’s silver foxes or paradigmatic domesticates such as cows, sheep, and goats, the selection against aggression that can result in the mammalian domestication syndrome is an *exogenous* selection pressure, normally due to the interaction with humans. However, according to Wrangham, in the case of bonobos and humans, the selection for tameness that produced the domestication syndrome was an *endogenous* one due to intra-species dynamics. Within this framework, because the selection pressure was endogenous, humans and bonobos can be said to have *self*-*domesticated*. Human and bonobo domestication was a process of *self*-*domestication*.^22^

Various details of Wrangham’s hypothesis need to be questioned. For example:

- Wrangham places emphasis on executions and it unclear whether this emphasis is justified. Boehm (2001) suggests that anti-alpha behaviors other than executions have also had an important evolutionary impact.^23^
- Wrangham claims that female coalitions are responsible for bonobo self-domestication but not for human self-domestication, where the selection pressures came from male-only coalitions (Wrangham 2019, p. 141). In contrast, Knight (1991) argues that human female coalitions—and the sex strikes through which women were able to obtain the cooperation of non-alpha males—have played a role in the process that made our ancestors able to achieve low levels of aggression. Moreover, Hrdy (2009) argues that women’s desires to share parenting responsibilities—and the resulting cooperative breeding practices—have contributed to the evolution of the kind of social tolerance observed in our species.^24^
- Boehm argues that big game hunting played a role in the evolution of anti-alpha tendencies in hunter-gatherers: when big game became an important source of protein, certain forms of egalitarian sharing became a safety net (i.e. variance reduction net); anti-alpha sanctioning made robust egalitarian sharing possible (cf. Boehm 2001, 2012). If this is right, selection against aggression in Late Pleistocenic hunter-gatherers may, in some circumstances, have been due (at least in part) to exogenous factors. Violent behaviors that disrupt group cohesion may result, in certain environments, in group-level failures; for example, depending on how animal sources of protein are distributed in the environment, they may result in the group’s inability to obtain enough meat protein regularly enough. The possibility of such group-level failures indicates that, alongside the endogenous selection pressures, there can also be exogenous selection pressures against aggression, and that two kinds of selection pressures can interact in interesting ways.

Although these issues cannot be explored further here, it is important to understand that the view that equates self-domestication with endogenous selection against aggression and the idea that humans might have become self-domesticated as a result of such selection are worth considering independent of what one might think of the details of Wrangham’s hypotheses.^25^ Humans niche-construct their own social environment, and sometimes they do so in ways that—directly or indirectly, and in interaction with non-social factors—select against aggression. Various forms of human agency, including some that Wrangham does not take into account properly, can produce endogenous selection against aggression.

Thinking about human domestication in terms of endogenous selection against aggression directs one’s attention to another way in which human agency has, at least potentially, contributed to human evolution. Exploring this process is important because it can be argued that the reduction of certain forms of violence has been crucial for the emergence of many of the complex ways in which humans cooperate.^26^^,^^27^

## Human domestication, social selection, and political selection

An individual may be disposed to interact cooperatively with individuals with certain specific traits (at least in certain circumstances); it may also be disposed to interact uncooperatively—or, in some cases, to avoid interactions—with individuals with other traits. Cooperative and uncooperative interactions come in many different forms and shapes. The interactions may be the result of implicit tendencies or they may be generated by explicit preferences, desires, or choices. They may be facilitated or impeded by the structure of the environment. The number of individuals involved in the interaction may vary. Any benefits or costs produced by the interaction may or may not be equally distributed among those involved. The term *social selection* can be used to refer to all selection pressures (and processes) generated by dispositions that affect the ways (cooperative or uncooperative) in which conspecifics interact.

Sexual selection can be seen as a special form of social selection generated by preferences for reproductive partners with certain traits. Kin selection, group selection, and “reciprocity” dynamics (direct or indirect) can also be seen as special forms of social selection in the sense defined here.^28^ Humans can form alliances for controlling and punishing individuals who might pose a threat. More generally, they can form alliances for controlling and punishing individuals exhibiting disliked traits or dispositions. According to Boehm, alliances of this sort in hunter-gatherer bands were a source of selection pressures during the Late Pleistocene. Selection pressures generated by punitive alliances are a form of social selection. Boehm observes:

Group members’ punitive actions can not only influence group life but also shape gene pools in similar directions […]. Therefore, we must ask if some limited purposeful element is actually creeping into a biological evolutionary process that, in theory, is supposed to be operating “blindly.” […] [T]wo totally unambiguous and potent practical examples of purposeful selection would be animal breeders and modern genetic engineers. We must also include members of discredited eugenics movements, for the Nazis knew exactly what they were trying to accomplish. All three consciously want to tamper with gene pools, and they all have some insight into what they are attempting. It’s with good reason that we don’t think of prehistoric hunter-gatherers as these kinds of active agents at all. Yet I shall propose that unwittingly their social intentions did affect gene pools in ways that were predictable, highly significant, and at least were guided by rather sophisticated immediate purposes that had to do with improving their quality of life. Prehistorically, I believe that this provided a special “focus” to the process of human social selection, a focus that derived from the very consistent practical purposes of the actors. (Boehm 2012, p. 16).

When they punish band members who behave in ways that are widely disliked within the band, hunter-gatherer rank-and-file coalitions do not have eugenic purposes: they are not aiming to improve the genetic stock of their society. In this sense, they are not engaging in Darwin’s methodical selection. In addition, associated band members are not (in general) aiming to deprive the disliked individual of the possibility to reproduce. Thus, hunter-gatherer coalitions are not engaging in Darwin’s unconscious selection. However, despite the fact that they do not result in “man’s selection,” hunter-gatherer coalitions can have evolutionary consequences. The evolutionary process discussed by Boehm can be usefully described in these terms:

1. In the population there are desires for specific political arrangements.
2. Such desires become widespread and result in various actions.
3. The actions have a transgenerational impact on the frequency and distribution of developmental factors (genetic and non-genetic) in the population.

A desire for a political arrangement can be said to be a *political desire*. Political desires are a special form of social preferences. They are desires about the kinds of interactions that should occur within one’s community, about who should interact with whom, and in what ways. They may concern the distribution of resources accessible to the community or the costs that group members need to pay in certain contexts. We can call *political actions* the behaviors motivated by political desires; and we can call *political selection* the kind of selection generated by political desires and the resulting actions. Political selection is a special form of social selection. Political selection can, in some circumstances, result in political arrangements that match the content of the political desires that produce it:

4. The actions—through their impact on the frequency and distribution of developmental factors—make the desired political arrangements more likely to occur in the future.

Consider the political desire that alpha-type domineering tendencies are not present in one’s society. According to Boehm, this desire was common in Late Pleistocenic hunter-gatherer bands. Through the actions of rank-and-file coalitions, this desire made alpha-type domineering tendencies transgenerationally less prevalent. Or consider the desire that some forms of egalitarian sharing are routinely performed. According to Boehm, this desire made egalitarian sharing transgenerationally more prevalent in hunter-gatherers. Boehm suggests these transgenerational changes were partly due to changes in gene frequencies: as a result of social ostracism and, more generally, of the punitive interventions of rank-and-file coalitions, individuals with domineering tendencies or an unwillingness to share were less reproductively successful. However, arguably, cultural (non-genetic) processes were also important: because of social pressures, individuals with those tendencies were less culturally successful (i.e. less likely to be taken as sources of cultural learning by other individuals).^29^

Political desires may be instrumental or intrinsic. Rank-and-file foragers might initially realize that being dominated, for example, is bad for their own individual wellbeing and that they can improve their own individual lives by fighting together against domineering alpha types. Over time, the instrumentality of the desired political arrangement might fade, at least in part. Individuals might end up learning that certain political arrangements are desirable and need to be pursued regardless of any individual benefits they are likely to get from them. The ethnographic record suggests that the hunter-gatherer dislike of alpha-type tendencies is both intrinsic and instrumental: rank-and-file hunter-gatherers have a partial grasp of the individual benefits they get from anti-alpha political arrangements, but they also acquire the local moral ethos that classifies alpha-type dispositions as bad irrespective of any individual benefits. When a given political arrangement is desired both instrumentally and intrinsically, the motivation for pursuing this arrangement is enhanced, and so is the motivation for persuading others that the arrangement is desirable.

The hunter-gatherer case suggests that political desires can be stable enough at the population level to provide directionality (a “special focus”) to the evolutionary process. This does not require any foresight (cf. also Mesoudi 2018); it only requires that the relevant desires are – through cultural transmission and through what Boehm calls “the very consistent practical purposes of the actors”—transgenerationally available. According to Boehm, during the Late Pleistocene rank-and-file forager coalitions and the anti-alpha political desires underpinning such coalitions contributed to the evolution of human altruism and morality (cf. Boehm 2001, 2012; cf. also Mameli 2013). According to Wrangham, human male coalitions willing to execute violent individuals led to the reduction of certain forms of human violence (cf. Wrangham 2019). According to Knight, human female alliances led to novel kinds of social exchanges in the human species, including those that made cumulative symbolic culture possible (cf. Knight 1991). All these processes should be seen as instances of political selection.^30^

In the previous section, we discussed the view of self-domestication as due to endogenous selection against aggression. An alternative proposal is that self-domestication is the outcome of political selection, regardless of whether this form of selection brings about reduced levels of violence. Within this alternative framework, humans can be classified as self-domesticated simply because they have been transformed by political selection.^31^ We believe that this way of characterizing self-domestication is interesting because it draws attention to the role of political agency in human evolution. *Political agency*—understood as the kind of agency driven by political desires—can generate selection pressures against aggression; however, depending on the desired political arrangements and on the context in which the relevant desires operate, it can also generate other kinds of selection pressures. Despite the fact that the inspiration for both proposals derives from what is known about hunter-gatherer rank-and-file—male and female—coalitions, Wrangham’s conception of self-domestication as endogenous selection against aggression and the conception of self-domestication as political selection need to be kept distinct.

Political agency is not normally considered a source of selection pressures and, because of this, it is neglected in much of the literature on human evolution.^32^ The evolutionary impact that humans have had on their own niche and lineage includes, as a sub-category, the evolutionary impact of human political agency. It is important to explore the ways in which desires aimed at shaping the structure of interactions within one’s society have affected human evolution. In the future—due to science, technology and, more generally, social transformation—profound changes in alliance formation and in the individual and collective understanding of the consequences of human agency are likely to occur. Will this lead to new political desires? Will it lead to new instances of political selection and to new processes of self-domestication? Only time will tell.

## Conclusion

Four different ways of thinking about human domestication have been discussed. These different conceptions are linked by family resemblance. General debates on domestication—understood as a distinctive evolutionary process—are interesting in their own right. However, debates specifically about human domestication are of special interest because of their potential contribution to the study of the many roles played by human agency in human evolution.

Darwin’s way of dealing with the topic of human domestication draws attention to the issue of whether there has been artificial selection within the human species. Leach’s conception of human domestication draws attention to the evolutionary impact that stable human-made environments have had on human populations. Wrangham’s (and Belyaev’s) treatment of human domestication draws attention to how human-generated selection against aggression has influenced the human evolutionary trajectory. The view of human domestication as due to political selection draws attention to the ways in which political agency has shaped human evolution.

Many different forms of human agency exist. These can be divided into explanatory categories in various ways depending on one’s theoretical purposes. Currently, a complete inventory of the ways in which human agency has affected human evolution is not available. There is no complete account of the evolutionary impact of human agency. Debates on human domestication are a tool (among others) for making progress in this area. A better understanding of the evolutionary impact of human agency could become an important tool for humanity. This tool could, in principle, be used—collectively and democratically—to improve the human condition, or at least to postpone human extinction.^33^

## Footnotes

1 “The idea […] that the peculiarities of human evolution are due to man being a domesticated or, rather, a self-domesticated animal, has also made some impact on the thinking of anthropologists and biologists. Now, this theory obviously depends on the meaning one may give to ‘domestication.’ The evolution of animals and plants differs from that of wild forms chiefly because artificial selection partially replaces natural selection. The selection pressure is shifted from traits that favor the survival and reproduction of the population in the wild to those useful to the owner and the breeder. However, natural selection continues to operate in man even in environments created by his culture, and in any case, it is not yet replaced by artificial selection. Hence, we cannot speak of ‘domesticated’ man as we can speak of domesticated animals and plants. In fact, ‘domestication’ of man is too vague an idea to be scientifically productive” (Dobzhansky 1962, p. 196). Some have tried to build an analogy between the artificial selection of animals and plants and the kind of selection pressures generated by human culture, which also act on human beings. Dobzhansky uses this analogy to make sense of, and then dismiss, the notion of human domestication. We argue below (Sect. 3, note 10) that artificial selection is a special (and peculiar) kind of anthropogenic niche construction, and that equating domestication with anthropogenic niche construction would make the notion of domestication too broad. However, there are alternative ways of thinking about human domestication that Dobzhansky does not consider.

2 Discussions of human domestication—and the view that there are important differences between wild and domesticated species and that humans belong in the category of the domesticates—can be found in ancient texts. In *Enquiry into Plants* (I, 3), Theophrastus claims that the human is the most domesticated (ήμερος) living being: “ἥμερον δὲ καὶ ἄγριον δίκαιον καλεῖν ἀναφέροντα πρός τε ταῦτα καὶ ὅλως πρὸς τὸ ἡμερώτατον· [ὁ δ᾿ ἄνθρωπος ἢ μόνον ἢ μάλιστα ἥμερον.]” (Theophrastus 1916, pp. 28-29). In *Statesman*, Plato discusses some differences between domesticated and wild animals and proposes an analogy between statesmanship (the art of ruling) and herding (Plato 1925). Aristotle discusses wild and domesticated animals in *History of Animals* and in *Politics* (1254b), while arguing for the claim that some human beings are slaves by nature, he likens slaves to domesticated animals used for their bodily services (cf. Aristotle 1965, 1932; cf. also Varro, *On Agriculture* I, XVII, in Cato and Varro 1934). Lucretius describes the anthropogenesis as a process that crucially involved “mollifying” and “breaking the temper” of human beings. In his account, such a softening occurred as a result of a sequence of transformations that included sedentism, monogamy, the joint care of children, and the emergence of a sense of compassion and fairness: “Inde casas postquam ac pellis ignemque pararunt, / et mulier coniuncta viro concessit in unum/cognita sunt, prolemque ex se videre creatam, /tum genus humanum primum mollescere coepit. /ignis enim curavit ut alsia corpora frigus/non ita iam possent caeli sub tegmine ferre, / et Venus inminuit viris, puerique parentum / blanditiis facile ingenium fregere superbum. / tunc et amicitiem coeperunt iungere aventes / finitimi inter se nec laedere nec violari, / et pueros commendarunt muliebreque saeclum, / vocibus et gestu cum balbe significarent/imbecillorum esse aequum misererier omnis. / nec tamen omnimodis poterat concordia gigni, / sed bona magnaque pars servabat foedera caste; / aut genus humanum iam tum foret omne peremptum, / nec potuisset adhuc perducere saecla propago.” *De Rerum Natura* (V, 1011–1027) in Lucretius (1924), pp. 456–458.

3 Darwin distinguishes between more and less sophisticated forms of methodical selection. The line dividing “artless” cases of methodical selection and cases of unconscious selection is a thin one (cf. the discussion in Sterrett 2002).

4 Darwin also argues that, in contrast to what can be observed in species that are known to be domesticated, skulls and brains have become larger in the human lineage (Darwin 1871, Vol. I, p. 145). Increases in cranial and brain size in hominid evolution are nowadays an established fact (e.g. Bailey and Gearey 2009), but there is evidence of a reduction in cranial and brain size in the last 20,000 or 30,000 years (Henneberg 1998; Bednarik 2014; see the discussion in Hood 2014; Wrangham 2019).

5 Darwin also cites the Spartan practice of killing weak and malformed newborns, but he regards cases like this as evolutionarily marginal. This example appeared in the second edition of *The Descent of Man*: “In Sparta, also, a form of selection was followed, for it was enacted that all children should be examined shortly after birth; the well-formed and vigorous being preserved, the others left to perish” (Darwin 1874, Vol. I, p. 29).

6 In his book on animal and plant domestication, Darwin discusses the effects of the Napoleonic Wars on the average height of men in France. The high death toll of those wars fell disproportionately on taller men, as they were preferred as soldiers (Darwin 1868, Vol. II, p. 211). Darwin does not classify this as a case of artificial selection, either methodical or unconscious. The shorter men were spared from battle, but they were not preserved because they were most “prized”; on the contrary, they survived because they were less “prized” as soldiers. The impact on reproduction was indirect and not the result of a choice to allow the most “prized” individuals to reproduce.

7 In Darwin’s view, sexual selection has had an important impact on human evolution. However, in his opinion, female choice is important in birds but not in humans (Darwin 1871). It has been claimed that this is partly due to Darwin’s Victorian prejudices, which saw the powers of human female agency as greatly inferior to those of human male agency (cf. Richards 2017; but see for example the claims in Darwin 1871, Vol. II, pp. 371–375). To Darwin’s chagrin, Alfred Russell Wallace remained skeptical about Darwin’s sexual selection hypotheses, both those concerning humans and those concerning other species (cf. Wallace 1878, 1889). In some articles and books written after Darwin’s death, Wallace argued that female choice could potentially drive human evolution (possibly towards higher standards of morality) when women become economically independent. Economic independence would enable women to act freely on their sexual preferences, in contrast to what was the case in Victorian society. Wallace concluded that if one wants female-driven sexual selection to operate in humans, one needs to fight for socialism: “When we allow ourselves to be guided by reason, justice, and public spirit in our dealings with our fellow-men, and determine to abolish poverty by recognising the equal rights of all the citizens of our common land to an equal share of the wealth which all combine to produce,—when we have thus solved the lesser problem of a rational social organisation adapted to secure the equal well-being of all, then we may safely leave the far greater and deeper problem of the improvement of the race to the cultivated minds and pure instincts of the Women of the Future” (Wallace 1890, p. 337; cf. also Wallace 1892, 1913).

8 Darwin uses sexual selection to explain, among other things, the patterns of variability in the human species (i.e. the physical differences between “races”) and he claims there are similarities between such patterns and those observed in some domesticated animal breeds (Darwin 1868, 1871).

9 On niche construction, see Odling-Smee (1988); Odling-Smee et al. (2003); cf. also Lewontin (1983). As is made clear in the existing literature, the notion of niche construction is broad: niche construction theory deals with any kind of evolutionarily significant impact generated by the interaction between populations of organisms and their environment.

10 Zeder argues that niche construction theory provides tools for understanding the entire range of evolutionarily significant effects of human activities on the non-human populations that live in close proximity to human settlements. Anthropogenic niche construction can affect such populations in various ways, including: (1) selective pressures operating in the wild may be relaxed if, for instance, human intervention reduces the threat of wild predators for the non-human population; (2) other selective pressures may instead intensify (for instance, human activities can facilitate the transmission of infectious diseases to and within the non-human population); (3) genetic drift might produce significant effects if the non-human population is small as a result of human intervention; and (4) new phenotypic variants can emerge and become intergenerationally stable in the non-human population as a result of the ontogenetic impact of persistent human environments (cf. Zeder 2016, 2018; Smith 2016). On the basis of such possibilities, one could consider the view according to which domestication occurs whenever anthropogenic niche construction generates evolutionarily significant effects on a given species, humans included. However, this notion of domestication would be too broad, and it would not add anything to niche construction theory (see note 1). Nowadays, almost all species are affected evolutionarily by human choices and activities, either directly or indirectly.

11 Leach’s account implies that nomadic foragers, unlike other human populations, might not be domesticated (cf. Leach 2007). Brüne (2007) and Wrangham (2019) provide some discussion of the moral and political problems raised by accounts according to which some extant human populations are domesticated while others are not. There are also accounts according to which all human populations are domesticated but not all in the same way: some populations might for example be more domesticated than others. All such differences could, in principle, be used to dehumanize (or infra-humanize) certain human beings. The issue of intraspecific human variation in relation to (the degree of) human domestication is important but is not addressed in this article. On dehumanization (and infra-humanization), cf. Haslam and Loughnan (2014).

12 The same applies to other pests or parasites that have become “acclimatized” (to use Leach’s term) to human-made stable environments. It is unsurprising that Leach’s notion of “unconscious selection” is different from and broader than Darwin’s: “Changes brought about by unconscious selection can include (1) unintentional by-products of deliberate selection by humans, (2) the results of natural selection operating in a human-modified environment, (3) variations permitted by the relaxation of natural selection pressures, and (4) the outcomes of new selection pressures that are brought about by humans but of which they are largely unaware. While the first requires the existence of artificial breeding intentions, the remaining three cannot be ruled out in explanations of human morphological change. For example, catastrophic natural selection events such as the mud flows and dust storms that can follow repeated vegetation clearance affect human and animal survival alike. Technologies that reduce the mortality of weaklings and diseases that emerge in densely crowded settlements similarly influence both human and animal genetic fitness. When morphological changes are not genetic but environmentally induced, they can be expected to occur in many of the species voluntarily or involuntarily sharing that new environment” (Leach 2003, p. 359). Leach concludes that: “If it is accepted that the criteria of domestication encompass more than the products (and by-products) of conscious selection, then the possibility of human domestication must be reconsidered, and researchers in biological anthropology, archaeozoology, and animal behaviour would do well to share their findings on the mechanisms (genetic, epigenetic, and ecophenotypic) which underpin them” (Leach 2003, p. 359). Even though she deploys a different conception of domestication, Leach, like Darwin, argues that the domestication process has resulted in interesting cross-species similarities.

13 One case worth discussing concerns dog domestication. Some researchers argue that canine domestication began as soon as some canids were able to approach human settlements and exploit the opportunities that human activities offered to them (cf. Horard-Herbin et al. 2014; Morey 1994; Zeder 2012a, b). In such views, when humans started artificially selecting canids (i.e. when humans started making choices about which of the canids they interacted with would be allowed or encouraged to reproduce) these animals had already been partially domesticated by human-made environments. One complication for a theory like Leach’s is that these domesticating processes likely started before the spread of sedentism (cf. Prassack et al. 2020). Canids can follow nomadic hunter-gatherers when they move their camp from one location to another. Even before permanent settlements, canids’ association with human-made environments could become sufficiently stable to be evolutionarily significant. This suggests that what is crucial is not sedentism per se, but the stability and the cumulative potential of the association between the human-made environments and the target species. Permanent settlements are a robust and reliable way of generating associations of the right kind. However, the dog case shows that, strictly speaking, permanent settlements are not necessary. Niche construction theorists discuss the *ecological inheritance* of constructed niches (Odling-Smee et al. 2003; cf. also Avital and Jablonka 2000). The canine example indicates that some organisms were able to ecologically inherit human-made niches—and do so in a way that allowed for the accumulation of modifications—before the spread of sedentism.

14 In this context, the term *syndrome* is used simply to indicate that there is a set of traits that tend to co-occur. In the literature, there is also a discussion of domestication syndromes in plants, microbes, and other organisms (cf. Hammer 1984; Steensels et al. 2019). We do not deal with the non-mammalian versions of the domestication syndrome in this article.

15 This can be thought of as a refined version of the ancient idea that there are important similarities between humans and paradigmatic domesticated species. On “outcomes views” of domestication, cf. also Thomas and Kirby (2018).

16 The list is adapted from Hare (2017).

17 Belyaev’s results and the existence of the mammalian domestication syndrome are not uncontroversial (cf. Lord et al. 2020). Moreover, if the syndrome is real, there is a need to explain the co-occurrence of its components, not only in evolutionary (distal) terms, but also in ontogenetic (proximal) terms. More than one ontogenetic mechanism could, in principle, be involved (cf. Zeder 2018). Some believe that a unified ontogenetic explanation can be provided. In particular, Wrangham and collaborators claim that the joint occurrence of seemingly independent morphological, physiological, cognitive, and behavioral traits can be explained by focusing on neural crest cell migration and related mechanisms (Wrangham 2019; Wilkins et al. 2014). Work on ancient human DNA seems to support the hypothesis that these mechanisms have been the target of selection in our lineage after the split from Neanderthals and Denisovans (Theofanopoulou et al. 2017). If these mechanisms are conserved across mammals, the similarities across species can, in principle, be explained by an evolutionary process that has affected the mechanisms in the same way in all the species that have the syndrome (cf. Sánchez-Villagra et al. 2018; for additional details about the human case, cf. Zanella et al. 2019). Two matters need to be considered: (1) young mammals are, in general, more socially tolerant than adult conspecifics because they need to be docile to elicit the help of adult conspecifics and to avoid being rejected or attacked; (2) given how mammalian ontogeny works, tinkering with neural crest cells seems to be an efficient way to produce juvenilization (cf. Wrangham 2019; Wilkins et al. 2014). According to Wrangham, selection against aggression results in selection for juvenilization, which in turn results in changes in neural crest cell (and related) mechanisms. On this view, the process not only leads to juvenile tolerance being retained into adulthood, but also to many other traits that are not directly the target of selection. Further work is needed to determine if this account is correct. In this context, it is useful to mention that the view that humans are paedomorphic, and that this is evolutionarily significant, is not new; cf. Bolk (1926); Gould (1978).

18 For example, it is present in some of the ancient texts we mentioned in note 2.

19 Belyaev’s hypothesis is an instance of a more general view according to which the explanation for what is distinctive about human cognitive, emotional and social abilities is to be found primarily in selection pressures generated within our lineage by the human social niche (cf. Godfrey-Smith 2004; Sterelny 2007). According to this view, human evolution has been driven by a complex cascade of feedback loops due to the social impact of human agency (c.f. Whiten and Byrne 1997; Tomasello 1999; Dunbar 2003; Laland 2018).

20 Boehm’s views build on what Lee (1988) says on the “fierce egalitarianism” of foragers, and what Woodburn (1982) says on the “aggressive” and “assertive” egalitarianism of hunter-gatherers. Wengrow and Graeber point out that, very likely, Pleistocenic hunter-gatherers were not always egalitarian (cf. Wengrow and Graeber 2015; cf. also Wengrow and Graeber 2018). However, this is compatible with reverse dominance hierarchies being common—and evolutionarily consequential—in human Pleistocenic societies. The variation of social and political arrangements in foraging societies, as well as the social and political experimentalism of such societies, should in any case be acknowledged (cf. also Kelly 1995). Such variation is important for the formulation of hypotheses about why social arrangements with novel characteristics emerged during the Holocene (cf. Knauft 1991; Flannery and Marcus 2012; Scott 2018).

21 Wrangham (2018, 2019) makes a distinction between “reactive” and “proactive” aggression. The former is retaliatory, exercised upon provocation, while the latter is planned and exercised coldly in the pursuit of specific goals. This distinction plays a role in Wrangham’s hypothesis, but it is not relevant here.

22 In should be noted that Darwin and Leach do not use the term *self-domestication*. However, the selection pressures that they consider when they discuss the possibility of human domestication are also endogenous to the human species.

23 Pinker, Clark, and Morris discuss hypotheses about the mechanisms through which violence has been reduced in human societies during the Holocene (Pinker 2011, 2018; Clark 2009; Morris 2015). We cannot examine these hypotheses here.

24 On cooperative breeding, cf. Burkart et al. (2009). The selection pressures that allowed women (and children) to impose the role of alloparents onto males, for example, could have contributed—without any need for executions – to the reduction of forms of violence not compatible with efficient *allomothering*. Hrdy argues that the cooperative breeding hypothesis can also help explain the formation of extended exchange networks (Hrdy 2009, Chapter 1). On the evolutionary impact of women’s and children’s strategies, cf. Hrdy (1981). On female coalitions in hunter-gatherers, cf. Lewis (2002, 2017).

25 It should be noted that Boehm, Knight, Hrdy, Clark, and Morris do not explicitly mention human domestication. It is Wrangham who connects Boehm’s work—and thereby all theories about how human agency might have resulted in reduced levels of aggression—with the debate on human domestication.

26 One interesting version of the view that selection against aggression played an important role in the evolution of human cooperation can be found in Knight (1991). Knight explains how the elimination of certain conflictual tendencies could have played a role in the taking off of cumulative symbolic culture, a form of cooperative interaction that can generate many other forms of complex cooperation. Knight presents this thesis in conjunction with less robust hypotheses about the role of menstrual cycles in this process, which is probably the reason why his insights have been overlooked (cf. Knight 2010; Knight and Power 2011; Knight and Lewis 2017; cf. also Whiten and Erdal 2012). For the view that cumulative culture explains the unprecedented scale, complexity, and ecological success of human cooperation, see Henrich (2016); for theories concerning the mental abilities that underpin human cumulative culture, see Tomasello (2019); Gonzalez-Cabrera (2017); Heyes (2018); and Sterelny (2012). In this literature, the idea that the elimination of certain forms of primate violence made an important contribution to the evolution of human cooperation is either not mentioned or discussed only briefly.

27 If it is true that human ecological success is explained in part by selection against aggression, one should also ask the following question: how, if at all, have the bonobos benefited from reduced levels of violence? Arguably, self-domestication led to an increase in sexual interactions and sexual pleasure in this species. Bonobos use sexual exchanges to reduce tensions, both within groups and between groups. The higher levels of violence seem to make these goods unavailable to chimpanzees (Furuichi 2011; Clay and de Waal 2015; de Waal 1982). Bonobo social tolerance is, in any case, lower and less sophisticated than human social tolerance.

28 It should be noted that there is no agreed-upon definition of *social selection* in the existing literature and there are disagreements about how social selection can be related to sexual selection and other evolutionary processes. We are using the term broadly and these disagreements are not important for current purposes. For some of the relevant discussions, cf. West-Eberhard (1979, 2003), Flinn and Alexander (2007), Nesse (2007, 2009), Boehm (2008, 2012), Lyon and Montgomerie (2012), Roughgarden (2012), Prum (2017).

29 For the debate on the evolutionary significance of the inheritance of non-genetic developmental factors, cf. Griffiths and Gray (2001); West-Eberhard (2003); Mameli (2004); Richerson and Boyd (2005); Sterelny (2012); Richerson and Christiansen (2013); Laland (2018).

30 According to Wrangham (and Hare), punitive female coalitions in bonobos resulted in the reduction of certain forms of bonobo violence (Wrangham 2019; Hare et al. 2012). This could be an instance of political selection. The social dynamics observed in this species suggest that bonobos have political desires and that these desires can generate selection pressures. The lack of the linguistic and symbolic abilities that humans possess makes bonobos’ political desires much simpler than those found in humans. The same is true of the alliance formation abilities of bonobos, and of their understanding of the consequences of their collective actions (these factors are important in political selection because of the role of political actions). If one is interested in identifying simple (and non-human) forms of political selection, the bonobo case seems to be an excellent one to focus on.

31 The same could be said about bonobos; see the previous note.

32 Bagehot’s views (Bagehot 1872) have already been mentioned. Bagehot explicitly connects domestication and certain forms of political agency. His hypotheses are different from those that have been articulated here. Their explanatory potential deserves to be explored in future work.

33 Cf. Friedrich Engels, *Dialectics of Nature*, in Marx and Engels (1987), p. 461. In his writings, Engels does not discuss the topic of human domestication, but he formulates a set of hypotheses about how human agency has affected the evolutionary process. He does this in *Dialectics of Nature*—in the “Introduction” and in “The part played by labour in the transition from ape to man”—and in a different way in *The Origin of the Family, Private Property and the State* (1884, in Marx and Engels 1990). Engels’ specific hypotheses on human evolution are to a large extent outdated, mainly because of their pre-Weismannian (and pre-Galtonian) features: Engels—like Darwin—believed in the “transmission of acquired characteristics” (cf. Weismann 1885; Galton 1871). In spite of this, his methodological intuitions and his views on the relevance of the evolutionary sciences for projects of political transformation are worthy of renewed attention.
